# The 10 Commandments for Endoscopic Minimally Invasive Tricuspid Valve Repair

**DOI:** 10.1177/15569845241264571

**Published:** 2024-09-09

**Authors:** Mohsyn Imran Malik, Michael W. A. Chu

**Affiliations:** 1 Department of Cardiac Surgery, London Health Science Centre, ON, Canada

## 1. Establish Appropriate Indications for Tricuspid Valve Intervention

Tricuspid valve regurgitation (TR) is ubiquitous, more often secondary, commonly associated with other conditions and can be challenging to sort out which patients suffer from TR as opposed to those who live with incidental TR. As established by the American Heart Association (AHA) and European Society of Cardiology (ESC) guidelines, isolated tricuspid valve intervention is generally reserved for patients with symptomatic, refractory, severe TR or with signs of right ventricular dysfunction (Table 1).^1,2^ Decision making can be straightforward in patients with primary TR; however, in the much more common secondary TR, it can be difficult to establish a clear relationship with TR and symptoms, particularly in patients with significant comorbidities, severe pulmonary hypertension, and/or right ventricular dysfunction. More importantly, evidence remains uncertain that correction of TR alone in many of these patients provides significant symptom relief and improves patient survival and quality of life. In borderline cases, serial clinical assessment and echocardiography can help make the best attempts to provide an optimal “symptom to TR” correlation when assessing increasing TR and progressive symptoms and determining likelihood of symptomatic improvement with TR correction.

**Table 1.** Guidelines for Tricuspid Valve Intervention.^1,2^

2020 ACC/AHA guidelines for tricuspid valve intervention	2021 ESC/EACTS guidelines for tricuspid valve intervention
*Class 1* • Severe TR at time of left-sided valve surgery *Class 2a* • Primary severe TR and right HF • Secondary severe TR with right HF and annular dilatation without elevated PAP or left-sided disease • Progressive TR at time of left-sided valve surgery with annular dilatation >4.0 cm or right HF *Class 2b* • Asymptomatic severe TR with progressive RV dilatation or systolic dysfunction • Severe TR with right HF with prior left-sided valve surgery and absence of severe pHTN or RV dysfunction	*Class 1* • Severe symptomatic TS or asymptomatic TS at time of left-sided valve surgery • Severe TR at time of left-sided valve surgery • Symptomatic severe primary TR without severe RV dysfunction • Severe secondary TR at time of left-sided valve surgery *Class 2a* • Moderate primary TR at time of left-sided valve surgery • Asymptomatic severe primary TR and RV dilatation • Mild-moderate TR with dilated annulus >40 mm or >21 mm/m^2^ • Severe secondary TR with RV dilatation or symptoms, in absence of severe RV or LV dysfunction or severe pHTN *Class 2b* • Transcatheter treatment of symptomatic secondary severe TR considered inoperable

Abbreviations: ACC, American College of Cardiology; AHA, American Heart Association; EACTS, European Association for Cardio-Thoracic Surgery; ESC, European Society of Cardiology; HF, heart failure; PAP, pulmonary artery pressure; pHTN, pulmonary hypertension; RV, right ventricular; TR, tricuspid valve regurgitation; TS, tricuspid stenosis.

More often, TR is seen concomitantly with left-sided valvular disease, particularly mitral valve disease. AHA and ESC guidelines recommend tricuspid valve intervention in patients with severe TR, dilated tricuspid annulus >40 mm with less than severe TR, or with signs of right-sided heart failure.^1,2^ Several studies have attempted to provide more evidence-based metrics for determining the role of concomitant tricuspid valve repair at the time of left-sided valve repair. In 2022, the Cardiothoracic Surgical Trials Network randomized trial examined patients undergoing concomitant tricuspid valve repair for moderate or less TR in patients undergoing mitral valve surgery for mitral regurgitation.^3^ Results suggested benefit from a tricuspid valve repair in patients with moderate TR, primarily with a reduction in TR progression at 2 years. However, there was no benefit in patients with tricuspid annular dilatation alone. Interestingly, concomitant tricuspid repair was associated with a significant increase in pacemaker implantation (14.1% vs 2.5%) and prolonged hospital length of stay but no differences in survival, quality of life, or functional status up to 2 years. Longer-term follow-up is required to understand the late effects of concomitant tricuspid valve repair, and further investigation is warranted to optimize need for permanent pacemaker insertion.

Ultimately, we recommend repairing the tricuspid valve at the time of mitral valve repair when there is at least moderate TR and that annular dilatation alone should not influence decision making.

## 2. Have a Decision-Making Process for Surgical Versus Transcatheter Intervention

Perhaps the most important commandment is choosing which patients should have surgical repair versus transcatheter approaches for their tricuspid valve disease. We generally consider a surgical approach first, particularly in young (<70 years of age), active patients with an anticipated long life expectancy. Most patients present with concomitant valvular or coronary disease, especially mitral disease, that warrants surgical correction and thus would be best to have their tricuspid valve repaired at the same time. In addition, surgery may be better suited for patients presenting with complex anatomy, annular dilatation, previous or active infectious endocarditis, or pacemaker lead entrapment. The traditional indications and contraindications would likely make most of these patients eligible for a minimally invasive, endoscopic approach. However, many of the patients with isolated tricuspid valve disease present late with severe pulmonary hypertension, right ventricular dysfunction, hepatic congestion and fibrosis, and recurrent arrythmias. In addition, other comorbidities need to be considered to determine if the patient can tolerate general anesthesia, single-lung ventilation, and cardiopulmonary bypass. Transcatheter approaches may help mitigate some of the higher-risk features in patients with older age, severe frailty, chronic lung disease, end-stage renal dysfunction, end-stage liver disease, high-risk reoperation, and previous cerebrovascular disease.^4^

**Fig. 1.**
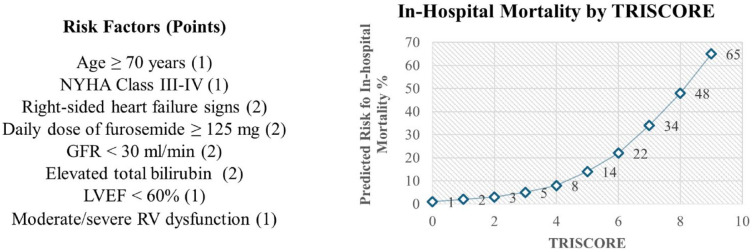
TRI-SCORE scoring rubric with correlated in-hospital mortality graphed by score. GFR, glomerular filtration rate; LVEF, left ventricular ejection fraction; NYHA, New York Heart Association; RV, right ventricle.

To aid in this decision making, the TRI-SCORE risk assessment tool for in-hospital mortality for isolated tricuspid valve surgery has been developed.^5^ This model considers many of the aforementioned risk factors, including age, right ventricular dysfunction, renal and hepatic function, and symptoms. A score between 0 and 9 points predicts in-hospital mortality with superior sensitivity and specificity when compared with EuroSCORE and EuroSCORE II. A score greater or equal to 6 points portends a high surgical risk and could prompt consideration from transcatheter options (Fig. 1).

Transcatheter tricuspid repair and replacement techniques have evolved considerably over the recent past and are an important alternative in higher-risk patients. While there have been many proof-of-concept studies of these devices, thus far, we have only 1 randomized controlled trial comparing tricuspid valve transcatheter edge-to-edge repair (TEER) with medical therapy: the TRILLUMINATE Pivotal trial. While there was no significant difference in death or rates of hospitalization for heart failure, the percutaneous tricuspid TEER did suggest some improvement in quality of life metrics, albeit on Kansas City Cardiomyopathy Questionnaire reporting without sham controls, and reduction in severity of TR at 30 days compared with medical therapy.^6^ These results present a reasonable option for patients who have prohibitive surgical risk, although future studies will be needed for long-term follow-up and comparison with MICS.

In summary, it is imperative to establish a decision-making tree for surgery versus transcatheter approaches. The decision should be formulated based on patient age, surgical anatomy, concomitant indications, patient comorbidities, and institutional expertise.

## 3. Surgical Approach: Endoscopic, Minimally Invasive, or Conventional Sternotomy?

Careful patient selection is also needed when deciding between endoscopic, minimally invasive (MICS) repair versus sternotomy. There is limited MICS evidence given a greater focus on mitral and aortic disease, rather than tricuspid valve repair over the past 2 decades. However, from our experience, there are few “absolute” contraindications, particularly in mature and well-established MICS programs. For surgeons early-on learning the steps of MICS tricuspid repair, we recommend straightforward patients with minimal comorbidities and a favorable body habitus. We perform a cardiac-gated computed tomography (CT) scan of the chest, abdomen, and pelvis with arterial and venous phase contrast preoperatively to assess for peripheral vascular access as well as chest cavity anatomy. Ideal patients will have a chest wall to mitral valve distance of <170 mm (as most patients in our experience require concomitant mitral valve surgery) and femoral artery size of approximately 6 to 7 mm at least. In patients requiring isolated tricuspid valve repair, it is extremely unusual to have a long chest wall to tricuspid valve distance; however, the CT should be examined carefully to look for any important chest wall deformities, such as pectus excavatum, which can compromise exposure with challenging right atrial retraction against the deformed sternum. In addition, caution should be exercised in patients with active pulmonary abscess or empyema, such as in patients who use injection drugs, where traversing an infected space may compromise the durability of the surgical repair.

The cardiac gated CT can also provide valuable information regarding coronary anatomy, which may ultimately change your surgical approach if coronary bypass is also required. Increasingly, CT is being used to also assess the degree of mitral annular calcification, with better sensitivity than echocardiography, which can be an important factor in repair versus replacement strategy in patients requiring concomitant mitral surgery.^7^ Lastly, the CT will provide information about hepatomegaly, which can lead to issues with exposure if there is significant elevation of the right hemidiaphragm.

Most importantly, the quality of the tricuspid repair should not be compromised because of the approach. If the patient requires a complex tricuspid reconstruction, such as patients with completely destroyed tricuspid valve from endocarditis (Supplemental Video 1) or a very dysplastic congenital tricuspid valve, we do not hesitate to perform the operation via sternotomy to achieve the best valve reconstructive results, if we feel the same results cannot be easily achieved endoscopically.

## 4. Patient Positioning and Operative Exposure Are Keys to Success

**Fig. 2.**
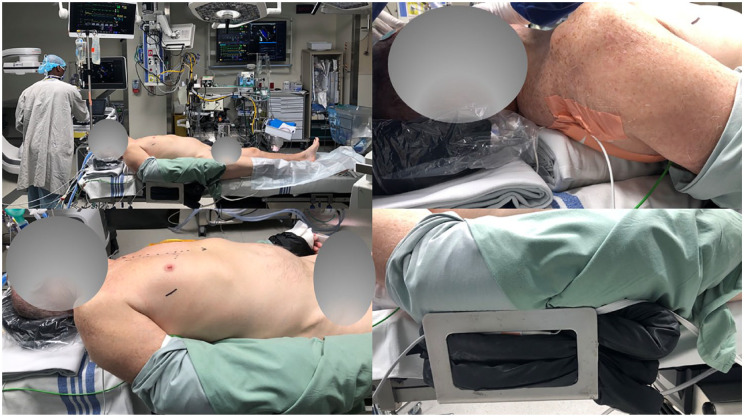
Intraoperative patient positioning for endoscopic minimally invasive tricuspid valve repair.

To facilitate ease of operation, optimizing patient positioning is essential. We have previously described the details of our positioning and operative exposure strategies.^8^ In brief, we prefer dual-lumen endotracheal tube intubation to allow for selective lung ventilation. We position the patient in a 20° left lateral decubitus with the right chest angled convex in a “C” shape to enable the widest intercostal interspace with the widest exposure to the tricuspid valve. The patient’s legs are placed in a neutral position to allow for ease of femoral access (Fig. 2).

We perform a double-stick technique to insert a percutaneous right internal jugular superior vena cava (SVC) cannula through the right internal jugular vein at the same time as central venous line insertion within the sterile surgical field. This also allows simultaneous chest port access development and femoral cannulation to optimize operative efficiencies.

**Fig. 3.**
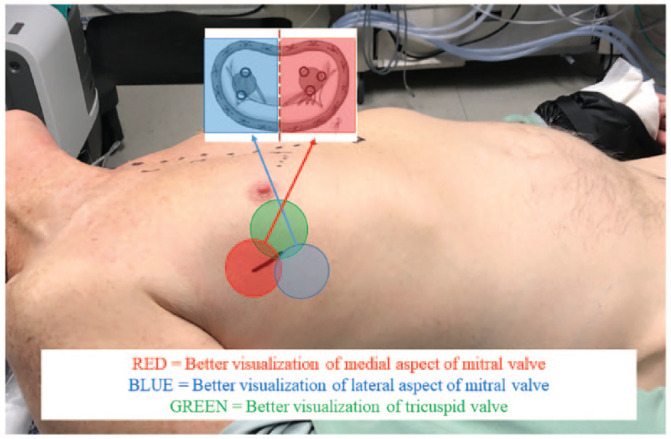
Right minithoracotomy angle of attack based on culprit valve lesion. Reprinted under STM Permissions Guidelines from Hage et al., *Ann Thorac Surg*, “Minimally invasive endoscopic mitral repair: how I teach it,” copyright 2022, volume 114, pages 35–39.

We routinely use a 3 to 4 cm port access incision and advocate using the CT to optimize the “angle of attack” from the endoscope and port to the tricuspid valve to provide best exposure.^8^ In general, we most commonly employ the fourth intercostal space and find that a more anterior port provides better access to the right atrium and tricuspid valve. A periareolar approach can also provide a nice cosmetic result for isolated tricuspid repair surgery. If the plan is for concomitant repair of both the mitral and tricuspid valves, we make our incision more superior–posterior if more medial disease of the mitral valve, versus an inferior–posterior incision if pathology of the valve is more lateral (Fig. 3).

**Fig. 4.**
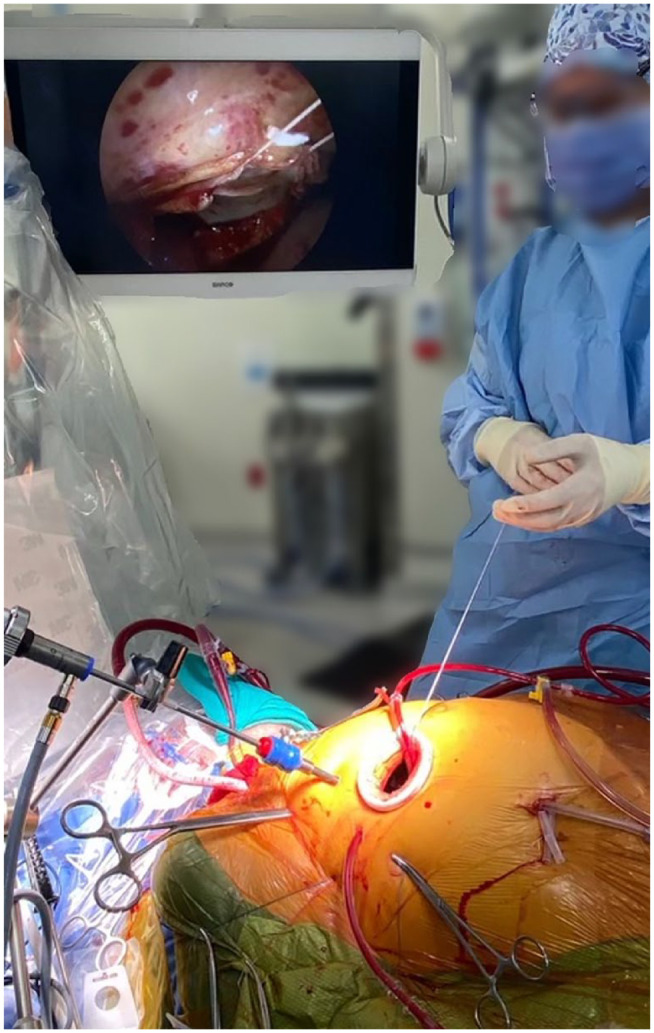
Intraoperative setup for endoscopic minimally invasive tricuspid valve repair via a right minithoracotomy incision. Reprinted under STM Permissions Guidelines from Hage et al., *Ann Thorac Surg*, “Minimally invasive endoscopic mitral repair: how I teach it,” copyright 2022, volume 114, pages 35–39.

Routine use of diaphragmatic and pericardial retention sutures can greatly help bring the heart closer to the port access. Unlike the mitral valve, the tricuspid valve can commonly be seen directly; however, we find that a 5 mm endoscope can greatly enhance visualization (Fig. 4). We generally prefer to open the pericardium and control the cava for tricuspid surgery; however, in reoperative surgery, the pericardium can be left intact and the right atrium opened before the heart is decompressed. This expeditious access can also help facilitate hemostatic closure of thin right atrial walls with the thicker pericardium attached to the right atriotomy.

**Fig. 5.**
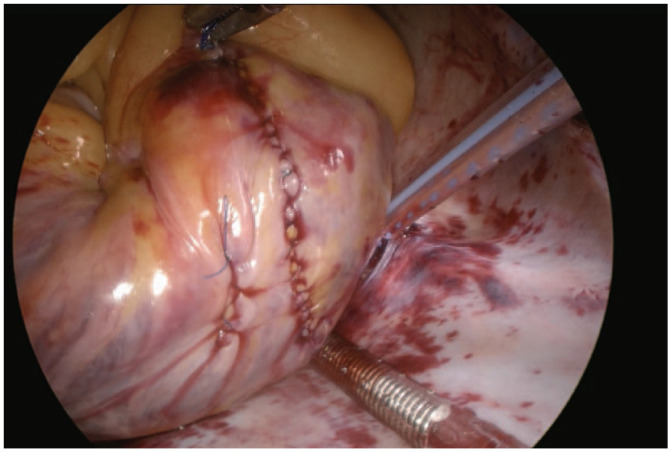
A vertical right atriotomy, after closure.

We commonly use a right vertical atriotomy incision for access to the tricuspid valve as we find it provides good exposure, is easily amenable to a hemostatic closure, and may be associated with reduced risk of atrial tachyarrhythmias (Fig. 5).

## 5. Follow the Important Principles of Safe Cannulation and Myocardial Preservation Strategies

While there are several options for cannulation and myocardial preservation, we utilize a similar approach for most of our MICS valve operations. We routinely employ percutaneous bicaval cannulation via right internal jugular vein (RIJV) and the common femoral vein (CFV). As previously described, RIJV access is obtained at the same time as central line placement. Generally, we want about 2 cm or more of separation between the central line and the RIJV cannula to avoid interaction. The RIJV cannula is advanced 5 to 7 cm at the level of the skin; it does not need to be advanced all the way into the SVC and can sit in the innominate vein with good upper body and cerebral drainage. We previously demonstrated in a randomized trial that routine percutaneous SVC drainage in MICS improves cerebral venous drainage and surgical visualization without increased risks or compromising surgical efficiency.^9^

Although we are well-versed in percutaneous arterial access, we still prefer to cannulate the femoral artery through an 8 mm Dacron side graft. We have demonstrated that it better preserves distal leg perfusion and believe that it also reduces the risk of retrograde aortic dissection. The CFV is cannulated in typical Seldinger fashion with usually a 21 or 25 Fr multiport cannula, depending on patient size. We advance the cannula to the level of the superior cavoatrial junction for initiation of cardiopulmonary bypass but withdraw it to just below the inferior cavoatrial junction after cardioplegic arrest. This enables optimal venous and cardiac drainage during cardioplegic delivery and then enables successful caval snaring. Some centers routinely use a single inferior vena cava (IVC) cannula with bicaval baskets, although we have found them less effective with regard to adequate drainage.

For myocardial protection, tricuspid valve repair can be performed on the beating heart, fibrillating heart, or arrested heart, particularly when just an annuloplasty ring is required. When more complex repairs are needed, including anterior leaflet patch augmentation or de novo leaflet reconstruction in endocarditis cases, we find it best to use cardioplegic arrest to ensure a still, motionless tricuspid annulus and right ventricle. As we perform most of our MICS tricuspid operations with mitral operations, we most commonly use aortic cross-clamping with del Nido cardioplegia delivered antegrade into the ascending aorta. In isolated tricuspid valve surgery, there is the option to perform surgery on a beating heart or fibrillating heart, which may be advantageous in certain scenarios, such as redo chest surgery with significant adhesions precluding cross-clamping or if there are concerns for heart block. We routinely flood our surgical field with CO_2_ to minimize intracardiac air, even on right-sided operations as well. For effective exposure of the tricuspid valve, we prefer placing caval snares in a similar manner to conventional methods used during sternotomy. The SVC snare can be clipped and left over the pericardium inside the chest out of the way. The IVC snare is usually externalized (Supplemental Video 2, Supplemental Video 3). Sometimes the posterior and septal annulus of the tricuspid valve can be dragged toward the IVC snare, and it can be helpful to unsnare the IVC snare. In these cases, it is very useful to use vacuum assist to prevent air lock and optimize venous drainage with an open IVC.

## 6. Choose the Appropriate Annuloplasty Ring/Band

Tricuspid valve repairs are most effective when a supportive remodeling ring annuloplasty is performed to address the commonly present annular dilatation, enhance leaflet coaptation, and optimize late repair durability. Although tricuspid ring sizing remains a matter of debate, we generally size the annuloplasty ring to the amount of anterior and posterior leaflet tissue to cover the tricuspid orifice area and optimize leaflet coaptation. This usually translates to ring sizes between 26 mm and 32 mm. There is no high-quality evidence to guide which ring or band is best. Observational studies have suggested that rigid tricuspid rings may be more prone to ring dehiscence.^10,11^ We have generally preferred incomplete, semirigid remodeling rings that preserve the Triangle of Koch and the conduction system. Tricuspid bands with even less circumferential coverage may have a theoretically lower risk of injury to the conduction system, but this remains to be proven. Newer iteration semirigid incomplete rings take a more 3-dimensional elliptical form rather than planar, to match the natural anatomy of the tricuspid annulus, potentially further mitigating stress on the conduction system and allowing more fluid and natural annular motion during the cardiac cycle.^12^

## 7. Consider Adjunct Repair Techniques

While most tricuspid repairs need only a ring to provide sufficient coaptation of the leaflets, some repairs may require additional work. In about 10% to 15% of our cases, we perform leaflet augmentation to increase coaptation surface area. Most commonly, this technique is used in patients with severely dilated annuli with insufficient leaflet area to ensure adequate coaptation or in patients with endocarditis with leaflet destruction. This is performed by incising the base of the anterior and posterior leaflets from the annulus and sewing in an autologous pericardial patch with locking expanded polytetrafluoroethylene (ePTFE) sutures. The patch is sized to the width of the anterior and posterior annulus with sufficient oversizing to account for contraction of the patch over time (Supplemental Video 1). Prior to placing our ring, we ensure there is enough leaflet area to cover the entire orifice. Less commonly, a patch may be required for the septal leaflet (endocarditis), in which cases careful bites must be taken to account for the risk of heart block.

In addition to leaflet augmentation, we occasionally create neochordae in a similar manner to the mitral valve to augment our repair. Unfortunately, is it much more difficult to premeasure neochordae for the tricuspid valve due to the variable anatomy of the papillary muscles and the dynamic nature of the right ventricle. Therefore, we use a freehand approach and estimate the length of the neochordae required using a saline test to pressurize the right ventricle. We place our ePTFE sutures twice through either the largest papillary muscle adjacent to the target leaflet or the moderator band if there is no suitable papillary muscle. We then pass both ends of the suture through the free margin of the target leaflet and test with saline to adjust our length before tying the suture down. In our experience, the length of the neochordae is more forgiving in tricuspid rather than mitral repair, allowing for a larger margin of error. Limited edge-to-edge repairs can also be last-resort options to salvage repairs with incomplete TR resolution and can be associated with reasonable durability as long as combined with ring annuloplasty.

## 8. When All Else Fails, Consider a Replacement

All possible attempts should be made to repair the tricuspid valve and avoid replacement. In general, tricuspid valve replacement is associated with worse short-term and long-term outcomes and survival compared with repair.^13^ Fortunately, only a small minority of patients may require replacement, such as in cases of severe leaflet loss or destruction from recurrent endocarditis, rheumatic disease, severe infiltrative disease, or cardiac masses.

We perform our replacements with interrupted 2-0 braided nonabsorbable pledgetted mattress sutures in an everting technique to minimize the risk of dehiscence or perivalvular leak. When placing our needle near the Triangle of Koch, we take our sutures into the leaflet only to avoid the conduction system. While mechanical tissue prostheses have been described, we strongly prefer the use of a bioprosthesis in the tricuspid position because of low-pressure gradients and excellent long-term outcomes.^14^ In addition, mechanical valves in the tricuspid position require targeting high international normalized ratios with warfarin due to the increased thrombotic risk in the setting of lower gradients. If patients eventually develop prosthesis failure, there is now the option of transcatheter valve-in-valve therapies that can be offered, which mitigates the need for future redo surgery.

## 9. Have a Plan for Dealing With Right Ventricular Dysfunction Perioperatively

Right ventricular dysfunction goes hand-in-hand with TR and can result in deleterious consequences after restoration of tricuspid valve competency. In addition, the common challenge of accurately assessing right ventricular function, especially in the setting of severe TR, compounds this problem further. Echocardiography provides a blunt estimate of right ventricular function, although septal and lateral wall global longitudinal strain may provide important prognostic value.^15^ Magnetic resonance imaging provides the best estimate of right ventricular function^16^ but still underestimates dysfunction in the setting of severe TR. Right heart catheterization can provide useful measurements for decision making, particularly in patients with severe pulmonary hypertension.

Preoperative optimization for right ventricular dysfunction and/or severe pulmonary hypertension can be trialed with inotropic-mediated diuresis and/or pulmonary vasodilators with reassessment of pulmonary pressures after treatment. If there is significant improvement of pulmonary pressures, demonstrating reversible pulmonary hypertension, then surgical repair may be suitable. However, if there is fixed severe pulmonary hypertension, surgical repair may be hazardous.

In cases in which the surgeon is struggling to come off cardiopulmonary bypass after repair due to right ventricular dysfunction, an algorithmic team-based approach between the surgeon and anesthesia is needed. Not uncommonly, the right ventricle may be temporarily impaired due to air down the right coronary and should recover with time. If the right ventricle is still impaired after deairing and reperfusion, the next steps are to optimize preload, contractility, and afterload. This includes judicious volume resuscitation; avoidance of hypoxia and hypercarbia, keeping the heart rate fast; the addition of inodialators such as milrinone or dobutamine; early initiation of vasopressin; and inhaled nitric oxide.

The early use of mechanical circulatory support can be helpful in patients with severely compromised right ventricular function or severe pulmonary hypertension. In our practice, we use a percutaneous right ventricular assist device (RVAD; ProtekDuo, LivaNova, London, UK) to temporarily support the right ventricle, placed after completion of the tricuspid valve repair, before weaning from cardiopulmonary bypass. We have demonstrated that early, prophylactic RVAD use in high-risk patients can reduce inotropic needs and enable successful sequential loading of the right ventricle with a newly repaired and competent tricuspid valve and is associated with good clinical outcomes.^17^ Alternative devices such as the Impella RP (Abiomed, Danvers, MA, USA) can also provide support up to 4.0 L/min of flow. If all else fails, rescue venoarterial extracorporeal membrane oxygenation can be considered but is often associated with poor outcomes.

## 10. Perioperative Management of Pacing

In tricuspid valve surgery, careful considerations of baseline and postoperative management of pacemaker needs should be well thought out. Preexisting transvenous pacemaker leads may present a problem when repairing the tricuspid valve and may in fact be the cause of increased TR. Lead impingement or entrapment of the leaflets and subvalvular structures may contribute to valvular insufficiency. Careful evaluation of the preoperative transesophageal echocardiography can help to evaluate this. Pacemaker lead removal preoperatively could be considered if pacing is no longer required or if the lead is infected or fractured. Alternate pacing could be considered temporarily or permanently. During tricuspid repair, the pacing wire should be mobilized to facilitate the repair. In the unlikely event that the lead cannot be mobilized, then the leaflet can be built around the lead; however, this makes future lead extraction hazardous and likely to destroy a repaired tricuspid valve and result in significant TR. Redundant leads can be incorporated into the anterior-septal commissure so that they sit within the native annulus and below the prosthetic ring. If there are issues with restriction of leaflet motion due to entrapment of the leads in the papillary musculature, the lead can be mobilized and tacked down to the moderator band or trabeculae. If the valve is being replaced, we prefer to remove the lead and plan for another long-term lead implant. We believe jailing the lead between the annulus and prosthesis should remain the last resort. Careful assessment of the lead function, durability, and placement can be made in collaboration with your electrophysiology partners to determine the best course of action.

When performing a tricuspid valve repair, steps need to be taken to minimize the risk of injury to the atrioventricular conduction system. As the atrioventricular node sits in proximity to the septal leaflet, the use of a partial ring with careful placement of annuloplasty sutures from 10 o’clock to 6 o’clock is crucial. While evidence is still lacking, some consider the use of overaggressive undersizing to be associated with an increased risk of pacemaker and may be considered in patients in whom you wish to avoid pacing. In addition, real-time assessment of conduction injury can be assessed when repairing the valve on a beating heart. However, whether the injury is reversible once discovered has yet to be determined.

Postoperative pacing needs should be anticipated, particularly with the recent recognition of the higher pacemaker needs after tricuspid repair. There is no evidence of increased pacemaker needs after MICS compared with sternotomy tricuspid repair. Patients with preexisting atrioventricular or intraventricular conduction disease, multiple valve surgeries, redo tricuspid valve surgery, tricuspid valve replacement, and concomitant ablation are at higher risk for permanent pacemaker.^18^ Intraoperatively, we place epicardial pacing wires on the diaphragmatic surface of the right ventricle in all patients. We place these while the heart is still empty on pump and prior to removing the cross-clamp as access to this area will be challenging after. In patients in whom exposure is impossible, such as redo cases with significant adhesions, we place a temporary transvenous wire.

Ideally, we try to avoid any wires being placed across the newly repaired or replaced tricuspid valve. In the past, we would place permanent epicardial wires at the time of surgery to avoid the need for a transvenous wire. However, we now have access to coronary sinus and leadless pacing, which prevents the need for a transvalvular wire and can be placed postoperatively before being discharged home. Leadless pacing is also an attractive option for patients who initially presented with or are at risk for endocarditis, as there is significantly less foreign material for infection to seed to.

## Conclusions

To consolidate these commandments, we have included a video of a patient undergoing concomitant endoscopic mitral and tricuspid valve repair at our center to demonstrate many of the principles discussed (Supplemental Video 4).
